# Different Dental Manifestations in Sisters with the Same *ALPL* Gene Mutation: A Report of Two Cases

**DOI:** 10.3390/children9121850

**Published:** 2022-11-28

**Authors:** Tamami Kadota, Marin Ochiai, Rena Okawa, Kazuhiko Nakano

**Affiliations:** Department of Pediatric Dentistry, Osaka University Graduate School of Dentistry, Osaka 565-0871, Japan

**Keywords:** hypophosphatasia, *ALPL* gene mutation, inherited disorder, early exfoliation

## Abstract

Hypophosphatasia (HPP) is an inherited disease caused by mutation of the alkaline phosphatase (*ALPL*) gene in an autosomal dominant or an autosomal recessive manner. The main symptoms of HPP are bone hypomineralization and early exfoliation of the primary teeth. Some of the mutations identified in autosomal dominant families are reported to have dominant negative effects. In addition, the penetrance can vary among patients with the same variant even within the same family, resulting in various phenotypes of systemic symptoms. However, differences in dental symptoms between patients with HPP and carriers with the same *ALPL* variant have not been reported. Herein, we report on two sisters who had the same heterozygous *ALPL* variant with dominant negative effects. The older sister had bone and dental symptoms and was diagnosed with childhood HPP. In contrast, the younger sister was a carrier with no bone and dental symptoms. It can be inferred that this phenomenon was caused by the difference in penetrance. This case revealed that carriers with the *ALPL* mutation may have no dental symptoms characteristic of HPP. Because HPP is sometimes progressive, it is very important to carefully monitor carriers to detect the possible onset of dental and systemic symptoms.

## 1. Introduction

Hypophosphatasia (HPP) is an inherited metabolic skeletal disorder caused by mutation of the alkaline phosphatase (*ALPL*) gene, which encodes tissue-nonspecific alkaline phosphatase (TNSALP) [1]. Any *ALPL* variant can affect the expression, folding, modification, and trafficking of the TNSALP protein, resulting in HPP in an autosomal dominant or an autosomal recessive manner [2,3].

Patients with HPP and bone hypomineralization have various systemic symptoms, such as rickets-like disorder growth hormone deficiency, craniotabes, craniosynostosis, blue sclerae, flail chest, costochondral enlargement, scoliosis, thickening of the wrists, knees, and ankles, bowing of the legs, lax ligaments, and hypotonia [4]. In addition, osteoporosis and frequent bone fractures are often observed at and after the onset of adulthood [5]. The dental manifestation of HPP is early exfoliation of the primary teeth before the age of four caused by the disturbed formation of root cementum [6,7,8].

HPP can be suspected based on the presence of bone hypomineralization and/or early exfoliation of primary teeth and is diagnosed using clinical symptoms and biochemical test results, especially low serum alkaline phosphatase (ALP) [9]. The serum ALP should be measured to determine whether age-specific normal values are met [10]. The International Federation of Clinical Chemistry and Laboratory Medicine (IFCC) reference method recommends that clinicians should suspect HPP when the serum ALP is less than 100 U/L in growing children [11]. Finally, the *ALPL* gene test is recommended to obtain a definitive diagnosis of HPP [12]. Although it has been reported that some carriers with the *ALPL* mutation may have mild systemic symptoms [13,14], there are few reports of dental symptoms in carriers. Herein, we describe different systemic and dental manifestations identified in sisters with the same *ALPL* gene mutation. The aim of this case report was to compare the dental symptoms of a patient with HPP with a carrier from the same family, and to underline the important role that dentists play in the management of patients with HPP and carriers.

## 2. Case Report

Two sisters two years apart in age were revealed to have the same *ALPL* mutation by genetic testing. Interestingly, each sister was found to have the *ALPL* mutation through different circumstances.

The older sister was referred to the Pediatric Dentistry Clinic of Osaka University Dental Hospital by a general dental practitioner for severe mobility of the primary anterior teeth at the age of six years and four months [8]. She had a general history of short stature, and a dental history of premature exfoliation of the mandibular bilateral primary incisors by the age of one. We suspected HPP based on her systemic and dental symptoms, and she was diagnosed with childhood-type HPP based on mild metaphyseal irregularities and a low serum ALP. She subsequently underwent *ALPL* genetic testing, which revealed the heterozygous *ALPL* mutation “c. 550C > T, p.Arg 184Trp in exon 6” (residual enzyme activity: 0.6 percent) [8].

The younger sister came to our clinic at the age of six years and one month because her mandibular right central primary incisor had exfoliated with the root remaining. Although she had no systemic symptoms, her parents suspected HPP as it is an inherited disorder and her sister had already been diagnosed with the condition. The serum ALP concentration measured with the Japan Society of Clinical Chemistry (JSCC) reference method was 306 U/L (normal range for a six-year-old girl: 460 to 1250 U/L), and plain radiography of the metaphysis was normal; thus, a diagnosis of HPP was not made based on the clinical findings. However, genetic testing revealed that she had the same type of heterozygous *ALPL* mutation as her older sister.

**Figure 1:**
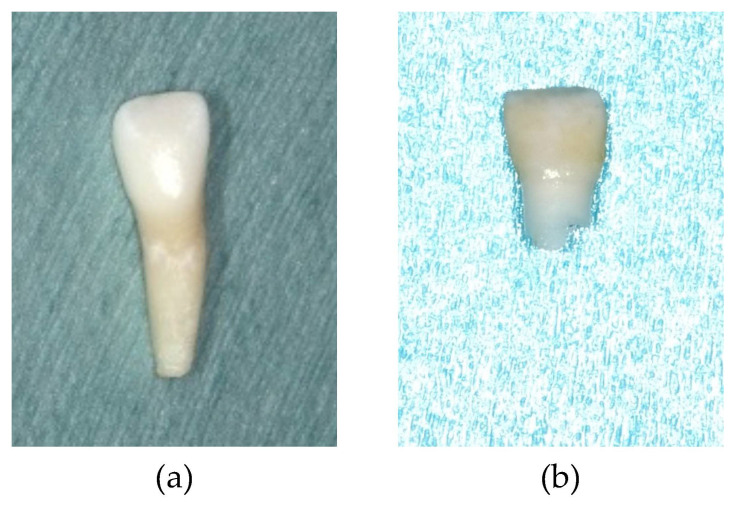
Exfoliated mandibular primary central incisors: (**a**) Older sister (seven years and three months); (**b**) Younger sister (six years and one month).

**Figure 2:**
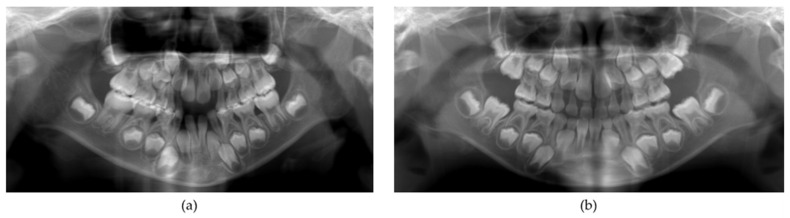
Panoramic radiography: (**a**) Older sister (seven years and three months); (**b**) Younger sister (six years and one month).

**Figure 3:**
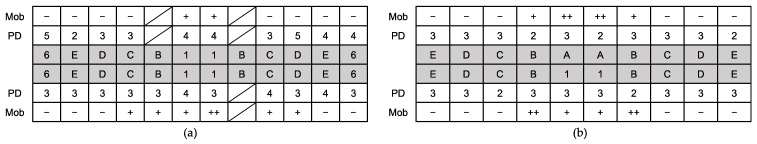
Periodontal pocket depth (PD) and mobility of teeth (Mob): (**a**) Older sister (seven years and eleven months); (**b**) Younger sister (six years and one month).

**Figure 4:**
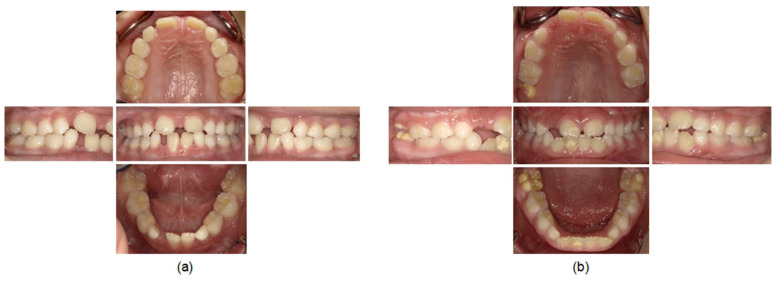
Intraoral photographs: (**a**) Older sister (seven years and eleven months); (**b**) Younger sister (seven years and two months).

The systemic symptoms in the older sister were a short stature at the age of four years, which was noted by a pediatrician. In addition, patients with HPP often have gait disturbance and she fell over frequently. The dental manifestations were the exfoliation of the mandibular bilateral primary central incisors at the age of eight years and–ten months without root resorption (Figure 1a). Periodontal examination revealed deep probing pocket depths and severe mobility of the remaining primary teeth at six years of age [8]. A panoramic radiograph revealed mild alveolar bone resorption in the posterior region (Figure 2a); similar findings were also observed after the permanent central incisors erupted (Figure 3a and Figure 4a).

In contrast to the older sister, the height and weight of the younger sister were within normal ranges from birth to the present. In addition, the younger sister did not have any bone symptoms or gait disturbance. Furthermore, only about one-fifth of the root of her mandibular right central primary incisor remained (Figure 1b), which is considered normal in the replacement of a primary tooth with a permanent tooth. A panoramic radiograph showed no alveolar bone resorption (Figure 2b). Periodontal examination revealed that the probing pocket depth was approximately 3 mm, which is deeper than the average pocket depth in children of 1 mm [15]; however, there was no abnormal tooth mobility (Figure 3b). When the younger sister was seven years and two months of age, she developed an amelogenesis disorder including enamel discoloration and defects of the permanent central incisors and first molars (Figure 4b).

In this case of two sisters with the same heterozygous *ALPL* mutation, the older sister was affected by HPP presenting as both bone and dental symptoms, while the younger sister was an asymptomatic carrier (Table 1). The older sister underwent periodontal treatment every month to prevent worsening periodontal conditions. Although the periodontal treatment was performed every three months during the primary dentition stage, the probing pocket depth of the permanent teeth remained more than 4 mm and mild mobility of multiple teeth was observed after the permanent incisors erupted; therefore, the treatment frequency was changed to monthly. In addition, she wore a mouthguard to prevent dental trauma when playing sports [16]; the mouthguard was continually adjusted or refabricated as needed. The older sister was expected to have insufficient space for eruption of the canine teeth, especially on the left side. In addition, we assumed that the jawbone may have shown poor growth because she was short of stature. Based on these factors, it can be inferred that she will experience teeth crowding in the future. Therefore, we have been monitoring the eruption of her permanent teeth and her jawbone growth in conjunction with orthodontists. Moreover, her growth and development are being monitored by a pediatrician, and she has received growth hormone treatment. In contrast, the younger sister has no dental symptoms characteristic of HPP; however, as dental symptoms may develop in the future, she undergoes regular checkups with particular attention paid to her periodontal conditions. Furthermore, her growth and development are being monitored by a pediatrician.

## 3. Discussion

HPP is an inherited disease associated with mutations in the *ALPL* gene that is located on chromosome 1p36.1-p34, is composed of 12 exons, and encodes TNSALP [17], which is essential for skeletal mineralization [1]. Three substrates of the TNSALP enzyme have been identified: inorganic pyrophosphate (PPi), pyridoxal-5-phosphate (PLP), and phosphoethanolamine (PEA) [18]. PEA is reportedly found in high levels in the urine of patients with HPP and may serve as a diagnostic marker for this disease [18]. *ALPL* mutations in patients with HPP are found at all exons, mostly comprising missense mutations, but also comprising frameshift and intron mutations due to the deletion or insertion of bases [1,13,14]. The *ALPL* mutation has been identified in more than 400 cases and listed in the *ALPL* gene mutation database [19]. In the present case, the *ALPL* variant detected in the sisters was c. 550C > T, which is a missense mutation. In addition, the residual enzyme activity was 0.6 percent, which is reported to have dominant negative effects [20].

HPP is inherited in either an autosomal dominant or an autosomal recessive manner. HPP caused by autosomal recessive inheritance is severe, while HPP caused by autosomal dominant inheritance is mild [21,22]. The frequency of severe HPP in the general population is estimated to be one per 6370 individuals in Europe [23], whereas that of mild HPP is expected to be much higher due to the number of patients with dominant forms of the disease [2,13,24]. Patients in families with autosomal recessive HPP are often compound heterozygous, with different mutations in each allele. Parents of patients with HPP are carriers with a mutation in only one allele and a low serum ALP concentration. Carriers may have mild symptoms but are usually asymptomatic. In contrast, patients from families with autosomal dominant HPP often have mild HPP, and only have a mutation in one allele [13,14]. In the present case, there was a history of HPP diagnosis in the paternal family members, but not on the maternal side. While the father did not undergo blood and genetic testing, these facts suggest that the father may also be an asymptomatic carrier of the heterozygous *ALPL* gene mutation and may have passed it on to the sisters.

HPP is classified into six types based on the time of diagnosis and symptoms: perinatal lethal, prenatal benign, infantile, childhood, adult, and odonto [12,25]. The systemic clinical manifestations seen in most patients with HPP include a rickets-like disorder and osteomalacia with an associated gait disturbance, but these manifestations have little influence on the life prognosis [26]. Patients with severe forms of HPP have life-threatening symptoms such as dyspnea due to pulmonary hypoplasia that occurs as a sequela to poorly mineralized ribs, vitamin B6-dependent seizures, and hypercalcemia [27]. Patients with perinatal lethal and infantile HPP often die at or soon after birth from respiratory insufficiency without treatment, while patients with childhood, adult, and odonto HPP have mild clinical symptoms [28,29]. The childhood type is characterized by skeletal deformities, short stature, and gait disturbance, while the adult type is characterized by stress fractures, thigh pain, chondrocalcinosis, and marked osteoarthritis [25]. The odonto type is characterized by premature loss of primary teeth, often without skeletal abnormalities [25]. Thus, there is variance between individuals in the severity and clinical manifestations of HPP, as well as the mutation types and inheritance forms of the *ALPL* gene. Some of the mutations identified in families with autosomal dominant HPP reportedly have dominant negative effects [13,30]. While the penetrance of autosomal dominant HPP is complete, it can be inferred that the dominant negative effects caused by *ALPL* mutation may result in incomplete penetrance [31]. The penetrance can vary between patients with the same variant, even within the same family, further contributing to the clinical variability among patients with HPP [32].

The sisters both had the c. 550C > T mutation, which is a missense mutation with dominant negative effects [33]. Despite having the same heterozygous *ALPL* mutation, the serum ALP concentration was below the lower limit of normal in the older sister, but was just barely above the lower limit of normal in the younger sister. In addition, a high PEA concentration was observed only in the older sister. It is very important to recognize that the serum ALP concentration fluctuates widely throughout the lifespan of an individual and must be compared with the age-specific reference range. The serum ALP concentrations are physiologically higher during infancy and adolescence, which are periods of rapid growth and bone metabolism [34,35]. Radiography showed metaphyseal irregularities only in the older sister. Children with HPP often have low bone mineralization, flared metaphysis, an area of radiolucency in the long bones at the extremities, and bowed and gracile bones [13,36]. Because only the older sister had short stature and gait disturbance, she was diagnosed with childhood type HPP [25]. The older sister had HPP with both bone and dental symptoms, while the younger sister was an asymptomatic carrier. It can be inferred that these phenomena were caused by the difference in penetrance.

The main dental manifestation of HPP is premature exfoliation of the primary teeth, and there are few reports of dental symptoms in carriers of the *ALPL* mutation. Premature loss of the primary teeth is caused by incomplete attachment of the roots to alveolar bone due to disturbed cementum formation [25]. In a previously reported case, histopathological examination of a mandibular primary incisor extracted at the age of three years and four months because of severe tooth mobility showed an almost complete absence of cementum [6]. Furthermore, immunohistochemical analysis of osteopontin, a marker of cementum, in TNSALP knockout mice (*Alpl−/−*) as a phenotype of infantile HPP revealed impaired cementum and alveolar bone formation [37]. Early exfoliation of primary teeth caused by cementum dysplasia may by differentiated from normal replacement based on the presence of minimal or no root resorption and tooth exfoliation at more than two years earlier than the average [7]. Disturbed cementum formation is likely to lead to exacerbation of periodontitis [38,39]. In the present case, the older sister had a probing pocket depth of over 4 mm. According to the classification of periodontal disease published jointly by the American Academy of Periodontology and the European Federation of Periodontology in 2017, a probing pocket depth over 4 mm is defined as periodontitis [40]. Although the younger sister’s probing pocket depths did not meet the definition of periodontitis, they were deeper than those found in normal children [13]. The root formation of the exfoliated primary tooth and the periodontal conditions suggest that the younger sister may also have slightly disturbed cementum formation. Thus, the changes in her periodontal conditions require careful monitoring. On the other hand, it is reported that patients with only dental symptoms, such as patients with odonto HPP, may sometimes develop bone symptoms after diagnosis [12]. The dental examinations of the younger sister revealed within the normal range, but may slightly resemble the characteristics of HPP. Although the younger sister had no bone symptoms, it is possible that she may develop symptoms of HPP in the future. Therefore, it is vital for the younger sister’s general condition that she be continually monitored by a pediatrician.

The younger sister also had amelogenesis of the permanent central incisors and first molars, which was considered molar incisor hypomineralization (MIH). MIH is a qualitative disorder of the enamel development that restrictively affects one or more permanent molars and incisors, with an unknown etiology [41,42]. Although there is no conclusive evidence regarding the etiological factors of MIH, the proposed causes include maternal illness or medication during pregnancy, birth defects, premature birth, prenatal illness, and prenatal administration of amoxicillin [43,44,45,46]. Based on previous reports, it was deduced that the *ALPL* mutation and MIH of the younger sister were unlikely to be related.

## 4. Conclusions

The present case report revealed the differences of dental and bone symptoms in individuals with the same *ALPL* mutation, even within the same family. As HPP is sometimes progressive, clinical symptoms may appear with growth in an individual initially considered to be an asymptomatic carrier. Therefore, it is important to implement early management of growth and development by medical professionals, as well as monitoring change in oral condition by a dentist.

## Figures and Tables

**Table 1:** Medical and dental symptoms of the two sisters.

Symptoms		Older Sister	Younger Sister
Medical symptoms	Short stature	+	−
	Low serum ALP	+	−
	High urine phosphoethanolamine (PEA)	+	−
	Metaphyseal irregularities	+	−
	Gait disturbance	+	−
Dental symptoms	Early exfoliation of primary teeth	+	−
	Probing pocket depth over 4 mm	+	−
	Mobility of teeth	+	−
	Hypomineralization of enamel	−	+

## Data Availability

Not applicable.
